# An interpretable consensus pipeline for candidate dopamine D2 receptor ligands

**DOI:** 10.1007/s40203-026-00749-7

**Published:** 2026-09-24

**Authors:** Sphamandla Mtambo, Hezekiel M. Kumalo, Cornelius Cano Ssemakalu

**Affiliations:** 1 Cell Biology and Drug Discovery Research Group, Department of Natural Sciences, Faculty of Applied and Computer Sciences, Vaal University of Technology, Vanderbijlpark, South Africa; 2 Drug Research and Innovation Unit, Discipline of Medical Biochemistry, School of Laboratory Medicine and Medical Science, University of KwaZulu- Natal, Durban, South Africa

**Keywords:** DRD2, Virtual screening, Interpretable machine learning, SHAP, Molecular dynamics, QSAR

## Abstract

Machine learning accelerates GPCR ligand discovery but often lacks interpretability and struggles to generalise to structurally novel chemical space. We present an integrated framework combining ensemble learning, SHAP interpretability, and complementary structure-based analyses to prioritise putative candidates for the dopamine D₂ receptor (DRD2). Models were trained on 1247 curated ChEMBL compounds using Bemis–Murcko scaffold-split validation. Feature ablation showed that Morgan fingerprints alone were sufficient for classification (AUC = 0.910), whereas combined physicochemical descriptors improved regression (R² = 0.606). Random Forest achieved scaffold-split classification AUC = 0.906 ± 0.016, and external PubChem BioAssay validation on non-overlapping compounds achieved AUC = 0.954. SHAP analysis identified nitrogen-containing substructural motifs consistent with Asp3.32 recognition, supporting the canonical DRD2 pharmacophore hypothesis. The pipeline recovered nine known DRD2-active compounds among the top 100 docked DrugBank candidates, corresponding to ≈ 14.6-fold enrichment over random selection. Virtual screening of DrugBank prioritised Roginolisib (DB18088), DB08010, and Bitopertin (DB12426) as putative DRD2 candidates. The near-zero correlation between ML predictions and docking scores (*r* = 0.090, *p* = 0.37, *n* = 100) supports their use as complementary evidence streams. Molecular dynamics simulations (200 ns) and MM-GBSA calculations indicated stable binding modes with geometries distinct from the reference ligand 8NU. By requiring agreement across complementary computational evidence streams, this consensus pipeline provides a reproducible framework for prioritising compounds in extrapolative GPCR chemical space. Experimental validation through radioligand binding and functional assays is required to confirm DRD2 activity.

**Supplementary Information:**

The online version contains supplementary material available at https://doi.org/10.1007/s40203-026-00749-7.

## Introduction

G protein-coupled receptors (GPCRs) represent the largest class of drug targets in modern medicine, targeted by approximately 34% of all approved drugs (Hauser et al. 2017). Among these, the dopamine D₂ receptor (DRD2) regulates motivation, reward processing, and motor control within the central nervous system. Dysregulation of DRD2 signalling is implicated in schizophrenia, Parkinson’s disease, bipolar disorder, major depression, and substance use disorders, making the identification of novel DRD2 ligands a therapeutic priority (Seeman 1987). Traditional drug discovery pipelines remain constrained by high development costs and substantial attrition rates, underscoring the need for efficient computational strategies to prioritise viable candidates prior to experimental validation (Kant et al. 2025; Scannell et al. 2012).

Virtual screening (VS) strategies generally leverage either target structure (SBVS) or known ligand information (LBVS) (Vázquez et al. 2020). Machine learning (ML) has increasingly advanced LBVS by modelling complex, non-linear relationships between molecular structure and bioactivity (Vamathevan et al. 2019). Recent advances in artificial intelligence have further expanded drug–target interaction prediction through deep learning models incorporating graph neural networks, attention mechanisms, and Transformer-based architectures, while related structure-based generative approaches have enabled interaction-aware molecular design (Huang et al. 2021; Nguyen et al. 2021; Zhang et al. 2025). These developments highlight the growing role of AI-driven approaches in ligand prioritisation and structure-guided drug discovery. Ensemble learning algorithms, such as Random Forest and XGBoost, are frequently employed to improve generalisation and mitigate overfitting (Neves et al. 2018; Svetnik et al. 2003; Taha 2025). However, two persistent limitations hinder the translational impact of ML-driven screening. First, predictive models often lack mechanistic interpretability, providing activity classifications without insight into the chemical determinants underlying those predictions (Rudin 2019). Interpretability facilitates rational optimisation by connecting statistical performance to chemical meaning. Methods such as SHAP (SHapley Additive exPlanations) attribute feature importance, connecting statistical performance with chemical interpretability (Lundberg and Lee 2017).

Second, ML model performance is highly dependent on molecular representation. Extended-connectivity fingerprints encode detailed substructural patterns, whereas physicochemical descriptors capture global molecular properties such as polarity and lipophilicity (Rogers and Hahn 2010). Systematic ablation studies comparing these representations remain scarce for GPCR targets, leaving it unclear whether substructural fingerprints, global descriptors, or their combination optimally encode the determinants of receptor–ligand recognition. Recent DRD2-related work using QSAR/deep learning (Won et al. 2025) and conformal prediction-guided docking (Luttens et al. 2025) has advanced ligand prediction and multi-target screening, though neither incorporates systematic feature-representation ablation or SHAP-based interpretability.

Furthermore, ligand-based prediction and structure-based plausibility are often evaluated independently, allowing high-scoring ML predictions to advance despite lacking physical complementarity with the target binding site. With the availability of high-resolution DRD2 crystal structures, it is now feasible to integrate ligand-based prioritisation with structure-based validation. Molecular docking provides an initial assessment of binding pose feasibility, while molecular dynamics (MD) simulations and molecular mechanics generalised Born surface area (MM-GBSA) free-energy calculations enable evaluation of conformational stability and thermodynamic favourability.

Here, we present an integrated, interpretable consensus pipeline for prioritising candidate DRD2 ligands from the DrugBank small molecule compound library. The novelty of this work lies not in the development of a new machine-learning algorithm or structure-based screening method, but in the systematic integration of established computational approaches into an interpretable, consensus-based prioritisation framework for DRD2 candidate ligands. By combining scaffold-aware ensemble learning, SHAP-based model interpretation, DrugBank virtual screening, molecular docking, molecular dynamics simulations, and MM-GBSA analysis, the workflow leverages complementary evidence streams to improve confidence when prioritising compounds from structurally diverse chemical libraries.

We hypothesise that ensemble ML models, optimised through systematic feature ablation and interpreted via SHAP analysis, can prioritise candidates while encoding chemically meaningful determinants of DRD2 recognition. Our workflow begins with the curation of a high-quality bioactivity dataset from ChEMBL, followed by Bayesian optimisation of ensemble classifiers and regressors. We evaluate molecular representations through ablation studies to identify the optimal combination of fingerprints and physicochemical descriptors. Prioritised hits undergo GNINA docking, followed by MD simulation and MM-GBSA free-energy calculations to assess binding stability. Applying this pipeline to DrugBank, we recover nine known DRD2-active compounds among the top 100 docked candidates (≈ 14.6-fold enrichment over random selection) and computationally prioritise three DrugBank compounds, Roginolisib (DB18088), DB08010, and Bitopertin (DB12426), currently characterised against unrelated targets, as putative DRD2 candidates with distinct simulated binding profiles. This establishes a methodological blueprint applicable to other data-sparse GPCR targets where screening libraries extend beyond previously characterised chemical space.

## Materials and methods

### Bioactivity data collection and curation

Bioactivity data for the human dopamine D₂ receptor were retrieved from ChEMBL (version 33, target CHEMBL217) using the ChEMBL web resource client (v0.10.9) (Mendez et al. 2019) on January 22, 2026. The query was restricted to compounds with reported IC₅₀ values from assays targeting human DRD2; Ki, EC₅₀, and all other activity types were excluded to ensure activity-type homogeneity across the training set. The complete ChEMBL target comprised 2,224 assay identifiers spanning 74 activity types; however, only standardised human DRD2 IC₅₀ measurements were retained for model development. Because ChEMBL IC₅₀ values originate from multiple experimental studies and assay formats, the curated dataset represents DRD2-associated bioactivity (potency) rather than a specific pharmacological mechanism such as agonism or antagonism. Compounds were therefore not stratified according to agonist, antagonist, or partial agonist activity. ChEMBL annotates this target as human DRD2 (CHEMBL217) and does not consistently distinguish between the D2 short and D2 long receptor isoforms; consequently, no isoform-specific analysis was performed.

Retrieved records underwent a ten-step curation pipeline implemented in RDKit (v2023.09.6) (Landrum 2023). Structures with missing SMILES were discarded; remaining structures were standardised using RDKit’s MolStandardize pipeline, comprising metal disconnection, largest-fragment extraction, functional group normalisation, charge neutralization, and canonical tautomer selection. Duplicate entries sharing the same canonical SMILES were identified after structure standardisation and aggregated by computing the median IC₅₀ and pIC₅₀ across replicate measurements, with the number of contributing assays recorded per compound. Compounds with undefined stereocentres were retained in their canonical SMILES form without stereocentre enumeration; stereochemical information present in ChEMBL records was preserved using isomeric SMILES throughout. IC₅₀ values were converted to molar units and transformed to pIC₅₀; ChEMBL’s pChEMBL field was used directly where available, with pIC₅₀ calculated as − log₁₀(IC₅₀ in M) otherwise. Records with pIC₅₀ outside the biologically valid range of 0–14 were discarded. Compounds with IC₅₀ outside 0.1 nM–1 mM or pIC₅₀ outside 3.0–10.0 were removed. Structural alerts were annotated using RDKit implementations of PAINS and Brenk (Brenk et al. 2008) filters; compounds triggering PAINS alerts were flagged but retained to avoid premature exclusion of chemically tractable actives. For binary classification, compounds with pIC₅₀ ≥ 6.0 (IC₅₀ ≤ 1 µM) were assigned to the active class (label = 1); those with pIC₅₀ < 6.0 were assigned to the inactive class (label = 0). The final curated dataset comprised 1,247 compounds, with 86.3% represented by a single IC₅₀ measurement after duplicate aggregation (median = 1 assay per compound; maximum = 11).

### Molecular feature representation

Two feature categories were computed for each compound. A set of 17 physicochemical descriptors was calculated using RDKit: exact molecular weight, MolLogP, TPSA, FractionCSP3, NumHDonors, NumHAcceptors, NumRotatableBonds, HeavyAtomCount, BertzCT, NumAromaticRings, RingCount, PEOE_VSA1, PEOE_VSA10, SlogP_VSA3, SMR_VSA10, BCUT2D_MWHI, and BCUT2D_LOGPHI, capturing complementary physicochemical, topological, and electrostatic surface area properties.

Morgan circular fingerprints (radius = 2, 2048 bits) were computed using RDKit, encoding extended-connectivity substructural environments equivalent to ECFP4 (Rogers and Hahn 2010). For the full feature set, descriptor vectors and fingerprint bitvectors were concatenated prior to model training.

### Machine learning model development

Three ensemble tree-based algorithms were implemented using scikit-learn (v1.6.1), XGBoost (v3.2.0), and LightGBM (v4.6.0) for binary classification and continuous regression. For classification, the target variable was the binary active/inactive label derived from the curated IC₅₀ threshold, representing DRD2-associated bioactivity. For regression, the target was the continuous pIC₅₀ value, providing an estimate of relative DRD2 potency. Neither model was designed to predict specific pharmacological functions such as agonism, antagonism, or inverse agonism.

The dataset was partitioned into training (75.3%, 939 compounds) and held-out test (24.7%, 308 compounds) sets using Bemis–Murcko scaffold-stratified splitting across 600 unique scaffolds with no scaffold overlap between partitions. This stratification achieves complete structural separation between training and evaluation sets.

The contribution of each molecular representation type was first assessed across three feature configurations: (i) Full (combined descriptors and fingerprints); (ii) Descriptors-only; and (iii) Morgan-only. Each configuration was evaluated independently for all three algorithms using default hyperparameters and the same scaffold-stratified train-test split. Performance differences were compared using bootstrapped 95% CI estimates on the held-out test set. Based on superior regression performance (R² = 0.606), the full feature set was selected for downstream model development.

Bayesian hyperparameter optimisation was subsequently performed using Optuna (v3.7.0) (Akiba et al. 2019) exclusively on the full feature set, with 500 trials per model-task combination. Scaffold GroupKFold cross-validation (20-fold) was applied during hyperparameter optimisation, preserving scaffold separation between training and validation folds throughout tuning. Final optimised hyperparameters are reported in Table S1. All cross-validation was performed exclusively within the training set. The held-out scaffold-stratified test set was not used during feature-set selection, hyperparameter optimisation, model selection, cross-validation, DrugBank screening threshold selection, SHAP interpretation, or applicability-domain threshold determination; it was reserved exclusively for a single final performance evaluation after all modelling decisions had been finalised. The full feature set (Morgan fingerprints combined with physicochemical descriptors) was defined a priori as the primary modelling configuration. Ablation analyses using Morgan-only and descriptor-only feature sets were subsequently performed to quantify the contribution of each feature type rather than to guide model selection.

Final model evaluation employed bootstrapped resampling (1,000 iterations) to generate 95% confidence intervals for all performance metrics. Classification metrics included accuracy, AUC-ROC, F1-score, precision, recall, average precision (AP), and Brier score. Regression metrics included R², RMSE, MAE, Spearman’s rank correlation (ρ), and Kendall’s τ.

### Model interpretability and validation

Model interpretability was assessed using SHAP (Lundberg and Lee 2017) with the shap Python library (v0.50.0). TreeSHAP was employed for exact Shapley value decomposition. SHAP values were calculated for all test set compounds and interpreted at global (mean absolute SHAP value rankings) and local (individual compound predictions) levels. Beeswarm plots were generated to visualise SHAP value distributions across features and to identify directional effects on predicted activity. Top-ranked features were interpreted with reference to the established DRD2 pharmacophore literature.

Y-randomisation tests were performed to exclude chance correlations. Binary activity labels were randomly permuted 100 times while keeping feature matrices intact. For each permutation, a classifier was retrained using the hyperparameters optimised for the original classification task and evaluated using 5-fold stratified cross-validation. This procedure was applied to all three feature configurations. A z-score was computed as the difference between the original and the mean permuted AUC, normalised by the standard deviation of the permuted distribution. A z-score > 3.0 (*p* < 0.001) was adopted as the criterion for rejecting the null hypothesis.

The applicability domain (AD) was assessed using a k-nearest-neighbour (kNN) Euclidean distance approach in standardised feature space. For each training set compound, the mean Euclidean distance to its five nearest neighbours (k = 5) was computed on z-score normalised feature vectors. The AD threshold was defined as the mean + 2σ of the training set kNN distances. Test set compounds exceeding this threshold were flagged as carrying additional uncertainty.

### Virtual screening of the drugBank library

The trained ML pipeline screened DrugBank (v5.1.13) (Wishart et al. 2018), comprising 10,041 compounds spanning approved, investigational, experimental, and other drug-group classifications as assigned by DrugBank. Canonical SMILES and molecular descriptors were computed for each DrugBank compound using the same descriptor-generation workflow applied to the ChEMBL training data.

Each compound was evaluated using an ensemble of three independently trained classifiers. The predicted active probability was calculated as the unweighted mean of individual model probabilities. Compounds were retained if they satisfied both criteria: (i) ensemble active probability ≥ 0.90; and (ii) predicted pIC₅₀ ≥ 7.0 from the best-performing regression model. These thresholds were selected to prioritise high-confidence predictions at the cost of recall, yielding a focused candidate set for subsequent structure-based screening. For the post-docking stage, the affinity-within-2.0-kcal/mol criterion anchors selection to the re-docked 8NU (Risperidone) reference pose; the CNN score requirement of ≥ 80% of the reference retains poses of comparable quality; and the secondary override rule, which admits compounds outperforming the reference affinity by more than 2.5 kcal/mol with CNN score at least equal to the reference, was included to accommodate structurally novel chemotypes for which ML predictions may be conservative. PAINS-flagged compounds were deprioritised but not automatically excluded.

A sensitivity analysis was performed by varying the primary ensemble probability threshold from 0.80 to 0.95 while holding the pIC₅₀ cutoff fixed at ≥ 7.0, to assess the robustness of the selected threshold and its effect on enrichment of confirmed DRD2-associated compounds in the pre-docking pool. Chemical-space coverage between the training set and the DrugBank screening library was assessed by counting unique Bemis–Murcko scaffolds in each, with intersection measured by exact scaffold-string matching. Two-dimensional visualisation used t-SNE (perplexity = 30) initialised from the first principal components of the standardised feature matrix.

### Molecular docking

Docking was performed using GNINA (v1.3.2) (McNutt et al. 2021), which incorporates a CNN-based scoring function to rescore poses generated by AutoDock Vina. Receptor preparation was performed using PDBFixer (v1.9) and OpenMM. The inactive-state human DRD2 crystal structure in complex with Risperidone (PDB ID: 6CM4, 2.9 Å) (Wang et al. 2018) was retrieved from the RCSB Protein Data Bank. The construct contained a T4 lysozyme fusion inserted into intracellular loop 3 and three engineered thermostabilising mutations (I122³·⁴⁰A, L375⁶·³⁷A, L379⁶·⁴¹A). All heterogens, including the co-crystallised ligand, ions, and water molecules, were removed. Missing residues and heavy atoms were added using PDBFixer, and hydrogens were assigned at pH 7.4 using the AMBER14 force field. Protonation states of all titratable residues, including Asp80 (Asp3.32) and histidines, were assigned using the AMBER14 default protocol. Crystallographic water molecules were removed, and no internal water networks were explicitly retained or modelled. Residue numbering follows PDB sequence numbering and the Ballesteros–Weinstein scheme for class A GPCRs. The receptor was energy-minimised using the AMBER14 force field and a Verlet integrator (time step = 0.001 ps) without positional restraints, and converted to PDBQT format using Open Babel. All docking calculations employed a single static receptor conformation without induced-fit modelling.

Ligands were converted to three-dimensional (3D) structures using MolScrub where available; otherwise, 3D conformers were generated with RDKit. Explicit hydrogen atoms were added using RDKit, and ligand geometries were optimised using the MMFF94 force field. No additional protonation-state prediction, tautomer enumeration, or charge reassignment was performed prior to molecular docking; therefore, ligands retained the protonation and tautomeric states present in the input structures.

The co-crystallised ligand 8NU (Risperidone) defined the docking box, centred on the ligand’s geometric centroid with a 4.0 Å extension in all directions using GNINA’s automatic box-generation procedure. This extension was selected to encompass the orthosteric binding pocket while allowing additional space around the reference ligand for compounds of varying size and shape. Docking exhaustiveness was set to 16; up to nine poses were generated per compound. Protocol validity was confirmed by re-docking 8NU, which reproduced the crystallographic pose with heavy-atom RMSD ≤ 2.0 Å.

For each compound, the top-ranked pose was selected based on the CNN affinity score. Post-docking prioritisation applied a two-stage selection. The primary stage required compounds to satisfy four reference-anchored criteria simultaneously, using 8NU (minimised affinity = − 10.801 kcal/mol; CNN score = 0.369) as benchmark: (i) GNINA minimised binding affinity within 2.0 kcal/mol of the reference ( ≤ − 8.801 kcal/mol); (ii) CNN pose quality score ≥ 80% of the reference (≥ 0.295); (iii) ensemble activity probability ≥ 0.75; and (iv) predicted pIC₅₀ ≥ 6.0. A secondary override admitted up to three additional compounds outperforming the reference affinity by more than 2.5 kcal/mol with a CNN score at least equal to the reference, accommodating structurally novel chemotypes for which ML predictions may be conservative. Compounds satisfying either criterion were ranked by GNINA minimised binding affinity, and the three compounds with the most favourable affinities were selected for molecular dynamics simulation.

### External validation

To assess model generalisation beyond the ChEMBL training distribution, an independent external dataset was constructed from PubChem BioAssay. Assay identifiers associated with human DRD2 were retrieved programmatically using PubChem PUG-REST queries based on UniProt accession P14416 and the DRD2 gene symbol, and concise bioactivity records were aggregated across the discovered assays. Records were restricted to IC50 measurements matching the activity type used for model training, and rows with non-numeric or non-positive potency values were excluded. PubChem compound identifiers were resolved to SMILES, compounds were structure-standardised using the same canonical SMILES procedure applied to the ChEMBL training data, and compounds already present in the training set were identified and excluded from evaluation. The trained ensemble, including applicability domain assessment recomputed using the same kNN-distance method and threshold definition as the training set, was applied to the remaining independent compounds in a single evaluation pass; no model retraining, threshold adjustment, or feature reselection occurred after this evaluation.

### Molecular dynamics simulations and trajectory analysis

All-atom MD simulations were conducted for the three top-ranked docking candidates and reference ligand 8NU, each in complex with DRD2 (PDB: 6CM4), using AMBER20 with GPU acceleration via pmemd.cuda (Case et al. 2020). The protein was modelled using the ff14SB (Maier et al. 2015). Ligand topologies and partial charges were generated using GAFF2 (He et al. 2020) in AmberTools20 antechamber, with AM1-BCC partial charges.

Each receptor-ligand complex was solvated in a truncated octahedral box of TIP3P water (Jorgensen et al. 1983) with 10 Å minimum solute-to-box-edge distance. Counterions were added to neutralise the system charge.

Energy minimisation proceeded in two stages: (i) solvent and ions minimised with positional restraints on protein heavy atoms (750 steepest descent + 1750 conjugate gradient steps); (ii) entire system minimised without restraints (50 steepest descent + 150 conjugate gradient steps). A non-bonded interaction cutoff of 12.0 Å was applied throughout.

Systems were heated from 0 to 300 K over 5 ps in the NVT ensemble using Langevin dynamics (collision frequency γ = 1.0 ps⁻¹) with positional restraints of 10 kcal/mol/Å² applied to the protein and ligand heavy atoms. This was followed by 500 ps of unrestrained NPT equilibration at 300 K and 1 atm. The heating phase was followed by extended NPT equilibration to ensure full system relaxation prior to production. Pressure equilibration was performed for 500 ps in the NPT ensemble at 300 K and 1 atm using Berendsen barostat (Berendsen et al. 1984) (pressure relaxation time τ = 2.0 ps), with all positional restraints removed. Production simulations ran for 200 ns per system at 300 K and 1 atm using the Berendsen barostat. Periodic image wrapping was applied throughout production. Coordinates were saved every 1 ps (500 steps × 0.002 ps), yielding 200,000 frames per system. Bonds involving hydrogen atoms were constrained using the SHAKE algorithm; long-range electrostatics were treated using particle mesh Ewald (PME) (Darden et al. 1993) with 12.0 Å real-space cutoff.

Trajectory analysis used cpptraj in AmberTools20 (Roe and Cheatham 2013). Structural stability was assessed via backbone RMSD of receptor relative to energy-minimised starting structure, per-residue RMSF of Cα atoms, radius of gyration, SASA, and protein-ligand hydrogen bonds at the active site.

Principal Component Analysis (PCA) was performed on Cα atoms of production MD trajectories to delineate the conformational landscape sampled by each receptor-ligand complex (David and Jacobs 2014). Analyses used cpptraj in AmberTools20. The mass-unweighted Cartesian covariance matrix of Cα atomic fluctuations was constructed and diagonalised to yield eigenvectors and eigenvalues. The first two eigenvectors (PC1 and PC2), capturing the largest proportion of total positional variance, were retained. The full trajectory was projected onto PC1 and PC2. Two-dimensional free energy landscapes were computed from PC1-PC2 projections:

$$ \Delta G{\text{ }} = - k_{{\mathrm{B}}} T{\mathrm{ln}}P(PC{\mathrm{1}},{\text{ }}PC{\mathrm{2}}) $$

where *k*_*B*_ is the Boltzmann constant, *T* = 300 K, and *P*(PC1, PC2) is the normalised probability density (Frenkel and Smit 2002). Free energy landscapes enabled the identification of dominant conformational basins and the assessment of the extent of conformational sampling.

### MM-GBSA binding free energy calculations

Binding free energies were calculated using MM-GBSA as implemented in MMPBSA.py in AMBER20 (Miller et al. 2012). Calculations were performed on snapshots extracted every 50 ps from the final 50 ns of each trajectory (1,000 frames per system). Per-residue energy decomposition identified individual amino acid contributions, focusing on key active site residues.

The binding free energy was computed as:

$$ \Delta G_{{bind}} = {\text{ }}G_{{complex}} - {\text{ }}G_{{receptor}} - {\text{ }}G_{{ligand}} $$

$$ \Delta G_{{bind}} = {\text{ }}E_{{gas}} + {\text{ }}G_{{sol}} $$

$$ E_{{gas}} = {\text{ }}E_{{int}} + {\text{ }}E_{{vdW}} + {\text{ }}E_{{ele}} $$

$$ G_{{sol}} = {\text{ }}G_{{GB}} + {\text{ }}G_{{SA}} $$

where *E*_*gas*_ represents gas-phase molecular mechanics energy (internal, van der Waals, and electrostatic contributions) and *G*_*sol*_ denotes total solvation free energy (polar Generalised Born and non-polar surface area terms). The conformational entropy term was omitted due to high computational cost and uncertainty, consistent with standard comparative MM-GBSA workflows.

## Results

### Dataset composition and curation

The ChEMBL database (target CHEMBL217) yielded 23,910 bioactivity records for the human dopamine D2 receptor. Following curation, 1,247 unique compounds were retained (5.2% retention rate). The curated dataset comprised 660 active (52.9%) and 587 inactive (47.1%) compounds. pIC₅₀ values ranged from 3.24 to 9.54 (mean ± SD: 6.23 ± 1.31). Drug-likeness assessment indicated that 82.8% of compounds satisfied Lipinski’s Rule of Five, while 58.0% were free of PAINS structural alerts. PAINS-flagged compounds were annotated but retained to preserve chemical diversity. Data completeness across descriptor fields averaged 98.7%.

For model development, Bemis–Murcko scaffold splitting generated a training set of 939 compounds comprising 529 active (56.3%) and 410 inactive (43.7%) compounds, and an independent held-out test set of 308 compounds comprising 131 active (42.5%) and 177 inactive (57.5%) compounds. No Bemis–Murcko scaffolds were shared between the training and test sets.

### Machine learning model performance

Three ensemble tree-based algorithms (Random Forest, XGBoost, LightGBM) were evaluated for binary classification and continuous pIC₅₀ regression. Hyperparameter optimisation was performed using Bayesian optimisation (500 trials) guided by 20-fold scaffold GroupKFold cross-validation. Model generalisation was assessed using a Bemis–Murcko scaffold-stratified held-out split (75.3% train / 24.7% test; 600 unique scaffolds with no overlap).

#### Classification performance

Under 20-fold scaffold GroupKFold cross-validation, LightGBM achieved the highest AUC (0.952 ± 0.028). On the scaffold-stratified held-out test set, Random Forest and LightGBM achieved equivalent AUC values (0.906 ± 0.016 and 0.904 ± 0.016, respectively), with XGBoost performing comparably (0.896 ± 0.016) (Table 1). The soft-voting ensemble attained AUC = 0.901 ± 0.017 with average precision (AP) = 0.872 ± 0.025. All models showed acceptable probability calibration (Brier scores 0.131–0.137). The Random Forest model achieved 81.1% accuracy on the held-out set, with precision favoured over recall for the active class (0.832 vs. 0.694), indicating a conservative profile suited to prefiltering candidates for downstream docking (Figures S1A-D).

**Table 1:** Classification performance on Bemis–Murcko scaffold-stratified held-out test set (*n* = 308 compounds; 600 unique scaffolds; no scaffold overlap)

Model	Accuracy	AUC (95% CI)	AP	F1	Precision	Recall	Brier
RF	0.811 ± 0.023	0.906 ± 0.016 (0.873–0.936)	0.877 ± 0.025	0.756 ± 0.032	0.832 ± 0.037	0.694 ± 0.041	0.131 ± 0.009
XGBoost	0.806 ± 0.022	0.896 ± 0.016 (0.862–0.925)	0.872 ± 0.024	0.772 ± 0.029	0.774 ± 0.038	0.771 ± 0.037	0.136 ± 0.013
LightGBM	0.811 ± 0.022	0.904 ± 0.016 (0.871–0.933)	0.876 ± 0.023	0.782 ± 0.027	0.766 ± 0.036	0.801 ± 0.034	0.137 ± 0.015
Ensemble	0.811 ± 0.023	0.901 ± 0.017 (0.867–0.932)	0.872 ± 0.025	0.778 ± 0.029	0.779 ± 0.037	0.778 ± 0.037	0.128 ± 0.012

Metrics: mean ± SD, bootstrapped 95% CI (*n* = 1,000 iterations). AUC = area under ROC curve; AP = average precision; F1 = harmonic mean of precision/recall; Brier score = mean squared probability error

#### Regression performance

Random Forest and XGBoost achieved comparable coefficients of determination (R² = 0.603 ± 0.042 and 0.604 ± 0.045, respectively), with the ensemble achieving the highest overall R² = 0.606 ± 0.043. Spearman’s rank correlation (ρ) for the ensemble was 0.765 ± 0.026. Residuals were approximately centred at zero across the central predicted pIC₅₀ range (~ 5.5–7.5), with deviations confined to the distribution extremes (Figure S2A-B).

**Table 2:** Regression performance on scaffold-stratified held-out test set (*n* = 308)

Model	*R*² (95% CI)	RMSE (pIC_50_)	MAE	Spearman ρ	Kendall τ
Random Forest	0.603 ± 0.042 (0.513–0.682)	0.751 ± 0.040	0.527 ± 0.031	0.758 ± 0.028	0.579 ± 0.027
XGBoost	0.604 ± 0.045 (0.504–0.684)	0.752 ± 0.043	0.530 ± 0.030	0.750 ± 0.028	0.576 ± 0.026
LightGBM	0.568 ± 0.048 (0.466–0.654)	0.782 ± 0.044	0.575 ± 0.031	0.747 ± 0.027	0.565 ± 0.026
Ensemble	0.606 ± 0.043 (0.520–0.683)	0.749 ± 0.041	0.532 ± 0.029	0.765 ± 0.026	0.586 ± 0.025

Metrics: mean ± SD, bootstrapped 95% CI (*n* = 1,000). R² = coefficient of determination; RMSE = root mean squared error (pIC₅₀ units); MAE = mean absolute error; Spearman ρ = Spearman rank correlation; Kendall τ = Kendall rank correlation

### Feature ablation study

Three feature configurations were evaluated: (i) Full (descriptors + fingerprints), (ii) Morgan fingerprints only, and (iii) RDKit descriptors only. On the scaffold-stratified test set, the Full feature set achieved ensemble AUC = 0.901 and R² = 0.606 (Table 3). Morgan fingerprints alone yielded marginally higher classification AUC (0.910) but reduced regression accuracy (R² = 0.578). Descriptors alone performed substantially worse (AUC = 0.848; R² = 0.419). The Full feature set was therefore used for downstream virtual screening, while the Morgan-only and descriptor-only models served as ablation analyses to assess the contribution of each feature type.

**Table 3:** Feature ablation results: Full (17 descriptors + 2,048 Morgan bits), Morgan-only, and Descriptors-only. CV = mean ± SD across 20-fold scaffold GroupKFold cross-validation pooled across three models; Scaffold = mean ± SD, 1,000 bootstrap iterations on held-out test set

Feature Variant	CV AUC	Scaffold AUC	CV *R*²	Scaffold *R*²	Scaffold RMSE
Full (Descriptors + Morgan)	0.949 ± 0.028	0.901 ± 0.017	0.712 ± 0.103	0.606 ± 0.043	0.749 ± 0.041
Morgan Fingerprints Only	0.947 ± 0.026	0.910 ± 0.016	0.694 ± 0.108	0.578 ± 0.047	0.775 ± 0.043
Descriptors Only	0.914 ± 0.040	0.848 ± 0.021	0.596 ± 0.123	0.419 ± 0.056	0.910 ± 0.040

### SHAP-based model interpretability

TreeSHAP was applied to the Random Forest classifier (scaffold-split AUC = 0.906) trained on the Full feature set. BCUT2D_LOGPHI emerged as the dominant global feature (mean|SHAP| = 0.0261). Dependence plots revealed a threshold effect at approximately 2.28 units, with compounds above this value contributing positively. *NumAromaticRings* was the strongest interaction variable, indicating that high-lipophilicity compounds with elevated aromatic ring counts received the strongest activity predictions.

The top five Morgan fingerprint bits were decoded to substructural patterns (Table S2). All five encoded nitrogen-containing motifs are consistent with the DRD2 pharmacophore centred on Asp3.32 coordination. Morgan_1295 (aryl amine motif) was the most selectively enriched substructure (4.16× enrichment in actives). Morgan_231 (tertiary amine in aromatic context) showed 2.88× enrichment. Morgan_1508 (diamine linker) showed 1.69× enrichment. Additional contributors included RingCount, BertzCT, and BCUT2D_MWHI (Figure S3A-H).

The Spearman correlation heatmap of the top 50 SHAP-ranked features (Figure S4) revealed that several nitrogen-containing Morgan bits (Morgan_1295, 231, 1508) were highly inter-correlated (ρ > 0.6), reflecting their shared role in encoding basic amine motifs central to DRD2 recognition. In contrast, the dominant physicochemical feature BCUT2D_LOGPHI showed only moderate correlations with these bits, highlighting its complementary contribution to capturing lipophilicity- and charge-distribution-driven effects not represented by substructural patterns.

### Virtual screening and candidate selection

Applying activity probability (≥ 0.90) and predicted potency (pIC₅₀ ≥ 7.0) thresholds yielded 253 compounds, of which 103 (41.4%) fell within the applicability domain (AD; δ = 68.15) and 146 (58.6%) represented extrapolations beyond the training distribution. Applicability domain status was tracked as a post-hoc indicator of prediction confidence rather than used as a pre-docking inclusion criterion; the top 100 candidates advanced to docking were selected by ensemble probability ranking alone, so that structurally novel compounds outside the training distribution were not systematically excluded from structure-based evaluation. The top 100 compounds ranked by ensemble probability were advanced to structure-based docking against the DRD2 crystal structure using GNINA with CNN-based scoring. Among these 100 compounds, 53 fell within the AD and 47 fell outside it. Re-docking of the co-crystallised ligand 8NU reproduced the experimental pose (heavy-atom RMSD ≤ 2.0 Å), validating the docking protocol. The median minimised binding affinity across the docked compounds was − 9.31 kcal/mol (Figure S5). All nine recovered known DRD2-active drugs fell within the AD (kNN distances: 22.47–64.41), consistent with their structural similarity to the training compounds. Candidates outside the AD were not excluded or down-weighted but were evaluated using the multi-stage consensus workflow to provide additional structure-based evidence where machine-learning predictions were less reliable. Among the three final candidates selected for molecular dynamics simulation, Roginolisib (DB18088) and DB08010 (experimental) fell outside the AD (kNN distances: 70.09 and 97.00, respectively), whereas Bitopertin (DB12426) fell within it (kNN distance: 62.93).

Post-docking refinement applied a two-stage prioritisation strategy combining reference-anchored docking criteria with machine-learning thresholds, supplemented by a predefined override for compounds exhibiting exceptionally strong structural evidence (Table 4). Of the 100 docked compounds, 96 satisfied the primary multi-criteria filter directly. Compounds satisfying the primary filter or secondary override were ranked by GNINA minimised binding affinity, and the three highest-ranked compounds were selected for molecular dynamics simulation. The highest-ranked compound by docking affinity, Roginolisib (DB18088), exceeded the reference ligand by 1.473 kcal/mol, below the 2.5 kcal/mol threshold required to trigger the secondary override. Consequently, the override criterion was not applied to any compound in this screening run. The top ten compounds ranked by GNINA minimised binding affinity are presented in Table 4. The structures, SMILES strings, charge states, and key physicochemical properties of the three final candidates are provided in Supplementary Table S3.

A sensitivity analysis varying the primary ensemble probability threshold from 0.80 to 0.95 showed that the pre-docking candidate pool decreased from 382 compounds at a threshold of 0.80 to 127 compounds at 0.95, with the default threshold of 0.90 yielding 253 compounds. Enrichment of confirmed DRD2-associated compounds, determined by canonical SMILES matching against the ChEMBL training set, increased progressively with threshold stringency from 8.5-fold at 0.80 to 19.1-fold at 0.95. The default threshold of 0.90 was retained as a pragmatic balance between enrichment and candidate pool size for downstream docking.

DrugBank compounds whose canonical SMILES matched compounds in the ChEMBL DRD2 training set (62 compounds) were intentionally retained throughout the screening pipeline to assess whether the consensus workflow could recover established DRD2 pharmacology. These compounds were therefore not excluded prior to virtual screening. However, the three final candidates selected for molecular dynamics simulation, Roginolisib (DB18088), DB08010, and Bitopertin (DB12426), show no exact canonical SMILES match to any compound in the ChEMBL DRD2 training set and were consequently prioritized as structurally novel computational hypotheses for DRD2 engagement.

The screening workflow recovered nine known DRD2-active drugs among the 100 compounds selected for docking (Table S4), comprising eight phenothiazine-class antagonists and one partial agonist. The background rate of DRD2-associated compounds in the full DrugBank library was determined by matching canonical SMILES against the ChEMBL DRD2 training set, identifying 62 confirmed DRD2-associated compounds among 10,041 DrugBank entries (0.62%). This yields an expected random recovery of 0.62 compounds per 100 docked, and the observed recovery of nine represents approximately 14.6-fold enrichment over random selection. As all nine recovered compounds are structurally related to compounds in the ChEMBL training set, this recovery demonstrates that the pipeline correctly prioritises established DRD2 pharmacophores rather than providing prospective validation on novel chemotypes. The near-zero Pearson correlation between ensemble-predicted pIC₅₀ and GNINA docking affinity across the 100 docked compounds (*r* = 0.090, *p* = 0.37) indicates an absence of significant linear association, supporting the interpretation that the two scoring approaches assess partly complementary properties (Figure S6).

Comparison of docking metrics between the nine pharmacologically established DRD2-active compounds and the remaining 91 shortlisted candidates revealed no statistically significant differences in GNINA minimised binding affinity (− 9.225 ± 0.599 vs. −9.381 ± 1.045 kcal/mol; t = 0.440, *p* = 0.661) or CNN pose quality score (0.553 ± 0.115 vs. 0.566 ± 0.158; t = − 0.236, *p* = 0.814). This finding indicates that, within the pre-filtered top 100 candidates, docking metrics alone did not discriminate established DRD2 ligands from other highly ranked compounds. This lack of discrimination is expected because all compounds had already satisfied stringent machine-learning and docking-based prioritisation criteria before this comparison and therefore do not represent an active–inactive benchmarking set.

**Table 4:** Top ten of the compounds passing post-docking filtering, ranked by GNINA minimised binding affinity

Rank	ID	Generic Name	Pred. pIC₅₀	Ens. Prob.	AD Distance	Affinity	CNN Score	CNN Affinity
1	DB18088	Roginolisib	8.243	0.988	70.09†	−12.274	0.607	7.607
2	DB08010	Experimental	7.047	0.991	97.00†	−10.924	0.640	6.351
3	DB12426	Bitopertin	7.379	0.978	62.93*	−11.089	0.352	7.166
4	DB19239	Tigozertinib	7.295	0.991	119.32†	−11.024	0.390	7.871
5	DB06525	Ganstigmine	7.877	0.984	95.56†	−10.981	0.772	7.245
6	DB11832	Crenolanib	7.415	0.997	85.70†	−10.966	0.711	7.868
7	DB15327	Abivertinib	7.760	0.996	104.92†	−10.895	0.418	7.905
8	DB02673	Experimental	7.679	0.996	75.51†	−10.801	0.664	7.518
9	DB07846	Experimental	7.626	0.999	84.39†	−10.790	0.602	7.041
10	DB14876	TAS-116	7.344	0.979	73.71†	−10.790	0.453	7.784
Ref	8NU	Risperidone	8.069	0.985	–	−10.801	0.369	7.439

Selection criteria: binding affinity within 2.0 kcal/mol of reference ligand 8NU; CNN score ≥ 0.295 (≥ 80% of 8NU reference); predicted pIC₅₀ ≥ 6.0; ensemble activity probability ≥ 0.75. Compounds exceeding the reference affinity by > 2.5 kcal/mol with CNN score ≥ reference were retained regardless of machine learning thresholds. Reference ligand 8NU included for comparison. Affinity, GNINA minimised binding affinity (kcal/mol); CNN score, GNINA convolutional neural network pose quality score (0–1); CNN affinity, CNN-predicted binding affinity (kcal/mol); Ens. Prob., soft-voting ensemble activity probability; Pred. pIC₅₀, ensemble regression-predicted pIC₅₀. AD Distance, mean kNN Euclidean distance in standardised feature space (k = 5; AD threshold δ = 68.15). Risperidone (8NU) was present in the ChEMBL training set with an experimental pIC₅₀ of 7.980; the predicted pIC₅₀ and ensemble probability shown for 8NU reflect internal consistency (prediction error = 0.089 log units) rather than out-of-sample prediction, since this compound was part of the model’s training data

*Within applicability domain. †Outside applicability domain

### Molecular docking interaction analysis

**Fig. 1:**
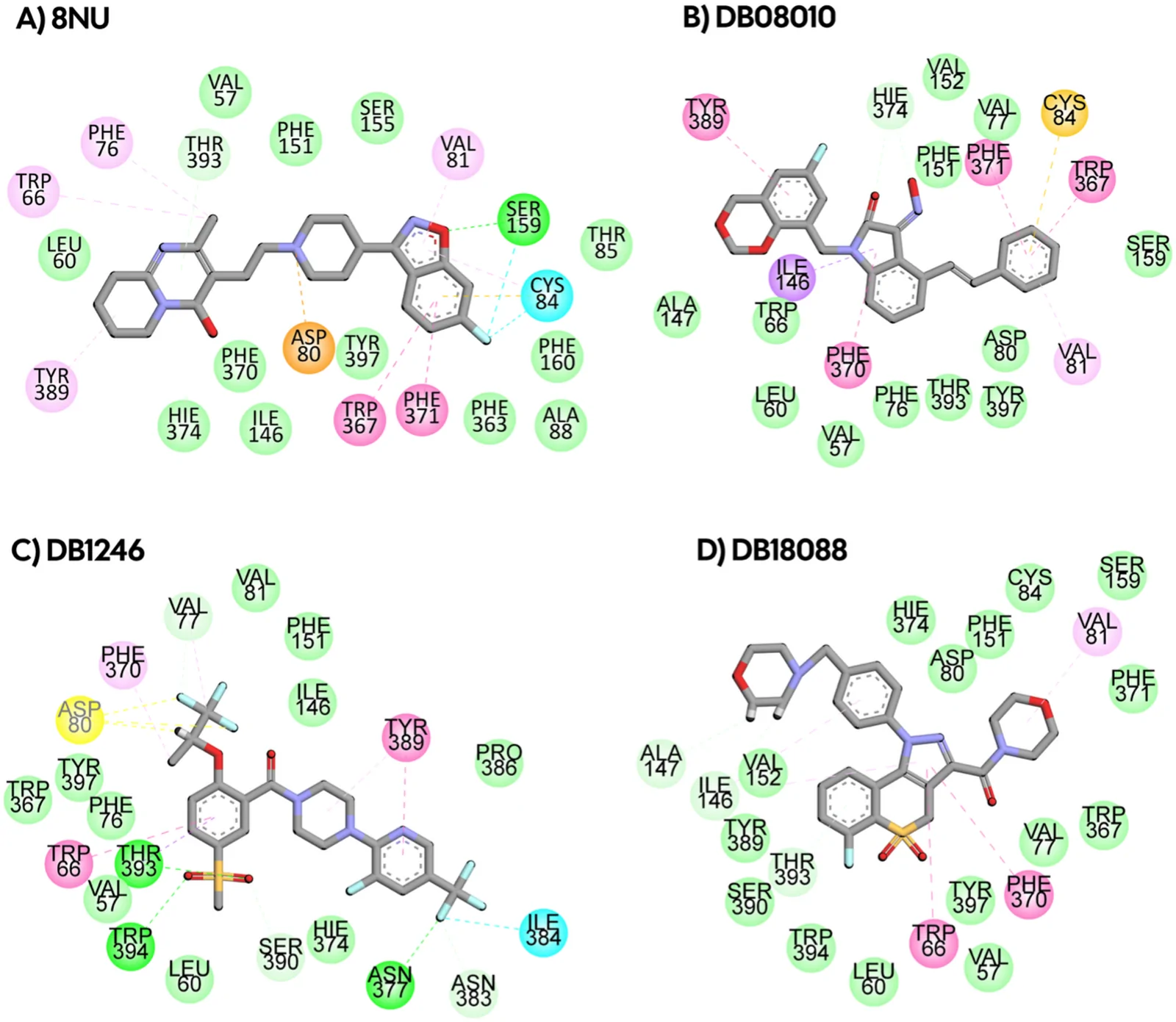
Two-dimensional ligand interaction diagrams for the four docked complexes at the DRD2 orthosteric binding site (PDB: 6CM4), generated using Discovery Studio Visualizer v.24.1.0.23298 (BIOVIA 2024). **(A)** Reference ligand 8NU. **(B)** DB08010. **(C)** DB12426. **(D)** DB18088. Residue colour coding: pink = hydrophobic contact; green = van der Waals contact; orange/yellow = ionic or salt-bridge interaction; cyan = water-mediated hydrogen bond. Dashed lines indicate directional hydrogen bonds (green) or halogen contacts (yellow). All interactions shown are within the energy-minimised docking pose prior to the MD simulation. A comparison between the docked and crystallographic binding poses of 8NU is provided in Supplementary Figure S10

Two-dimensional ligand-protein interaction maps are presented in Fig. 1. The reference ligand 8NU established the canonical DRD2 pharmacophore: ionic contact with Asp80 (Asp3.32), hydrogen bonds with Ser159, and extensive hydrophobic engagement (TRP367, PHE371, PHE370, PHE151, VAL81, HIE374, TRP66, PHE76, ILE146, and LEU60).

DB08010 adopted a deeply buried hydrophobic conformation dominated by contacts from Ile146. Asp80 was present within the interaction shell but showed only van der Waals contact. DB12426 displayed a polar binding mode, engaging Asp80 via direct ionic or hydrogen-bond interaction, along with additional polar contacts to Tyr389. DB18088 occupied a predominantly hydrophobic binding mode; Asp80 and His374 showed only non-directional van der Waals proximity. Across all complexes, the conserved hydrophobic core (Trp367, Trp66, Phe76, Ile146, Thr393) was engaged. The principal structural divergence was differential engagement of Asp80.

### Molecular dynamics: structural stability analysis

**Fig. 2:**
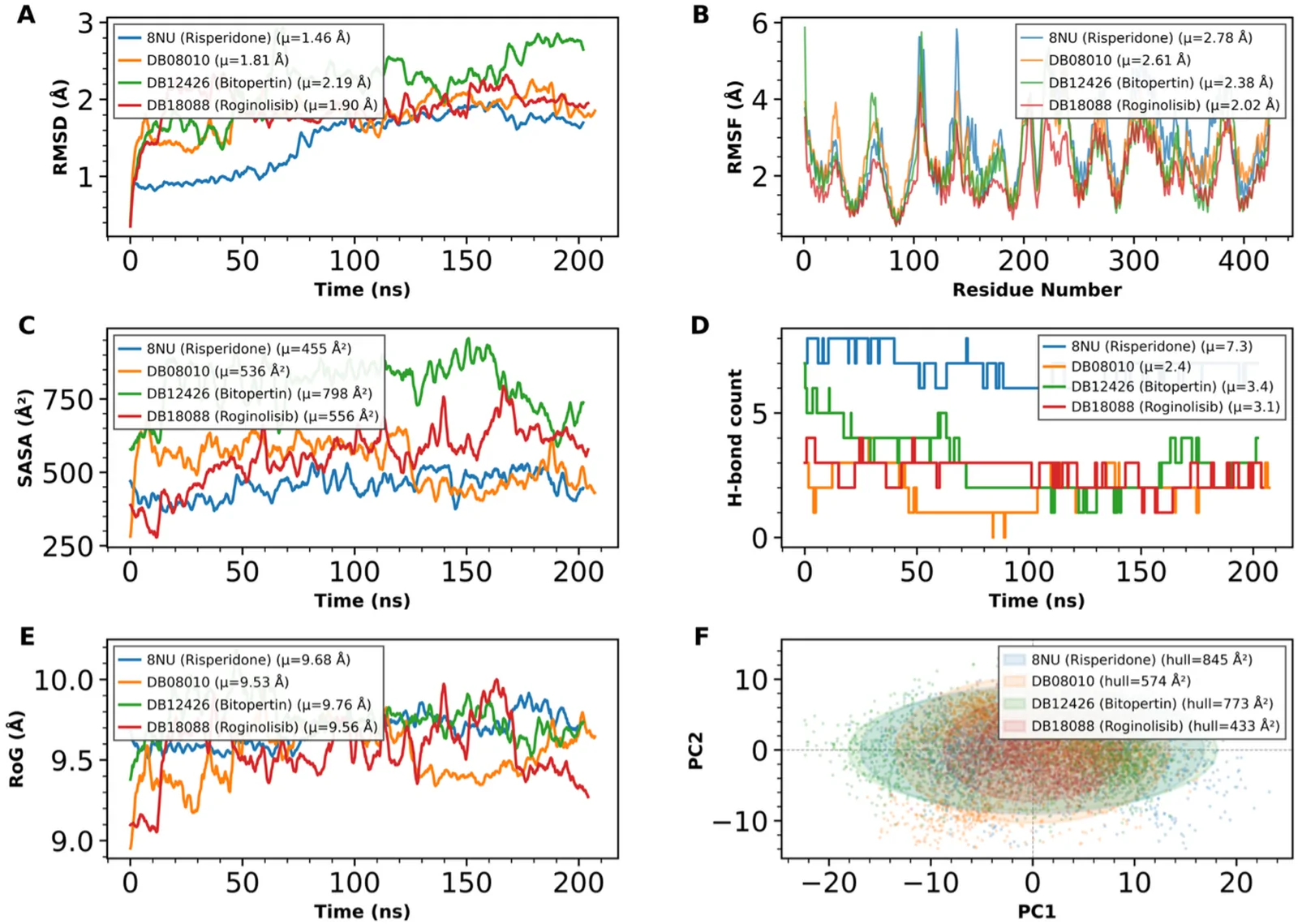
Molecular dynamics trajectory analysis across the four simulated complexes (200 ns production runs). All metrics were computed for active-site residues within 5 Å of the bound ligand in the energy-minimised structure, except per-residue RMSF **(B)**, which was computed across all Cα atoms. **(A)** Active-site RMSD (Å) versus simulation time relative to the energy-minimised structure. **(B)** Per-residue RMSF (Å) across the full protein. **(C)** Active-site solvent-accessible surface area (SASA, Å²) versus simulation time. **(D)** Active-site protein-ligand hydrogen bond count per frame. **(E)** Active-site radius of gyration (RoG, Å) versus simulation time. **(F)** PC1-PC2 conformational projection of active-site Cα atoms. Legend values (µ) represent trajectory means

Each candidate complex and the 8NU reference system were subjected to a 200 ns all-atom explicit-solvent MD simulation. Trajectory analysis focused on active-site residues within 5 Å of the bound ligand (Table 5; Fig. 2).

The reference ligand 8NU exhibited stable binding (active-site RMSD = 1.46 ± 0.39 Å). Among candidates, DB18088 produced the lowest active-site RMSD (1.90 ± 0.27 Å). DB08010 showed moderate stability (1.81 ± 0.27 Å), while DB12426 displayed the highest mean active-site RMSD (2.19 ± 0.45 Å). Protein-ligand RMSF values were similar across all systems (range 1.53–2.34 Å).

Solvent-accessible surface area (SASA) of the active site diverged substantially. 8NU exhibited the lowest mean SASA (455 ± 63 Å²). DB12426 displayed elevated mean SASA (798 ± 101 Å²), indicating increased solvent exposure. DB08010 (536 ± 87 Å²) and DB18088 (556 ± 108 Å²) showed intermediate values. Hydrogen bond analysis revealed that 8NU maintained the highest mean number of active-site hydrogen bonds (7.33 ± 1.58), while candidates showed reduced counts (2.37–3.44).

Principal component analysis of Cα atomic fluctuations revealed distinct conformational sampling patterns (Fig. 2F). DB18088 and DB08010 occupied compact regions of the PC1-PC2 conformational subspace. DB12426 displayed a broader distribution. Convex hull areas: 8NU = 845 Å²; DB08010 = 574 Å²; DB12426 = 773 Å²; DB18088 = 433 Å².

Ligand-centred trajectory analysis further supported stable retention of the docked poses throughout the aqueous-phase simulations (Table S5). Mean ligand RMSD remained below 2.2 Å for all complexes, although Bitopertin (DB12426) exhibited the largest positional deviation (2.174 ± 0.370 Å; final-frame RMSD 2.804 Å). Hydrogen-bond occupancy remained high for all ligands (92.1–100%), indicating persistent receptor interactions throughout the simulations. Ligand RMSF likewise showed greater flexibility for DB12426 than for the reference ligand 8NU.

**Table 5:** Molecular dynamics trajectory metrics (mean ± SD, 200 ns production run)

Metric	8NU	DB08010	DB12426	DB18088
RMSD (Å)	1.46 ± 0.39	1.81 ± 0.27	2.19 ± 0.45	1.90 ± 0.27
RMSF (Å)	2.10 ± 0.80	2.10 ± 0.65	2.34 ± 1.13	1.53 ± 0.49
RoG (Å)	9.68 ± 0.12	9.53 ± 0.19	9.76 ± 0.15	9.56 ± 0.22
SASA (Å²)	455 ± 63	536 ± 87	798 ± 101	556 ± 108
H-bonds	7.33 ± 1.58	2.37 ± 1.38	3.44 ± 1.67	3.11 ± 1.28
PCA hull area (Å²)	845	574	773	433

Metrics computed for active-site residues within 5 Å of bound ligand in energy-minimised structure, except RMSF (computed across all Cα atoms). RMSD = root mean squared deviation from energy-minimised structure (Å); RMSF = root mean squared fluctuation (Å); RoG = radius of gyration (Å); SASA = solvent-accessible surface area (Å²); H-bonds = mean protein-ligand hydrogen bonds per frame; PCA hull area = convex hull area of PC1-PC2 conformational projection (Å²)

### External validation

An independent dataset was assembled from PubChem BioAssay for human DRD2 (UniProt P14416) to evaluate model generalisation beyond the ChEMBL training distribution. Aggregating IC_50_ records across associated BioAssay entries yielded 1,576 harmonised records with usable potency values (827 active, 749 inactive at the pIC_50_ ≥ 6.0 threshold). Of these, 1,329 (84.3%) matched compounds already present in the ChEMBL training set by canonical SMILES and were excluded, leaving 247 independent, non-overlapping compounds for evaluation. Applicability domain analysis, recomputed using the same kNN-distance procedure as the training workflow, reproduced the expected threshold (δ = 68.83); 223 of 247 external compounds (90.3%) fell within the applicability domain.

In this locked single-pass external evaluation, the ensemble classifier achieved an AUC of 0.954 and an accuracy of 0.899, comparable to and numerically higher than the internal scaffold-split held-out test performance (AUC = 0.901; accuracy = 0.811) (Table 6). The regression ensemble achieved an R² of 0.515 and an MAE of 0.597 pIC_50_ units. These results indicate strong external classification performance and moderate quantitative potency prediction on independently sourced PubChem BioAssay compounds, while also highlighting the substantial overlap between public bioactivity resources for a well-studied target such as DRD2.

**Table 6:** Comparison of DRD2 model performance on the internal Bemis–Murcko scaffold-split test set and an independent external PubChem BioAssay validation set

Metric	Internal test	External PubChem BioAssay
Compounds evaluated (n)	308	247
AUC	0.901	0.954
Accuracy	0.811	0.899
Regression R²	0.606	0.515
MAE (pIC₅₀)	0.532	0.597
Within AD (%)	95.1	90.3

Reported AUC and accuracy correspond to the raw ensemble classifier, while R² and MAE correspond to the pIC₅₀ regression ensemble. Applicability domain (AD) membership was assessed using the recomputed DRD2 kNN-distance applicability domain threshold (δ = 68.83)

### MM-GBSA binding free energy

**Fig. 3:**
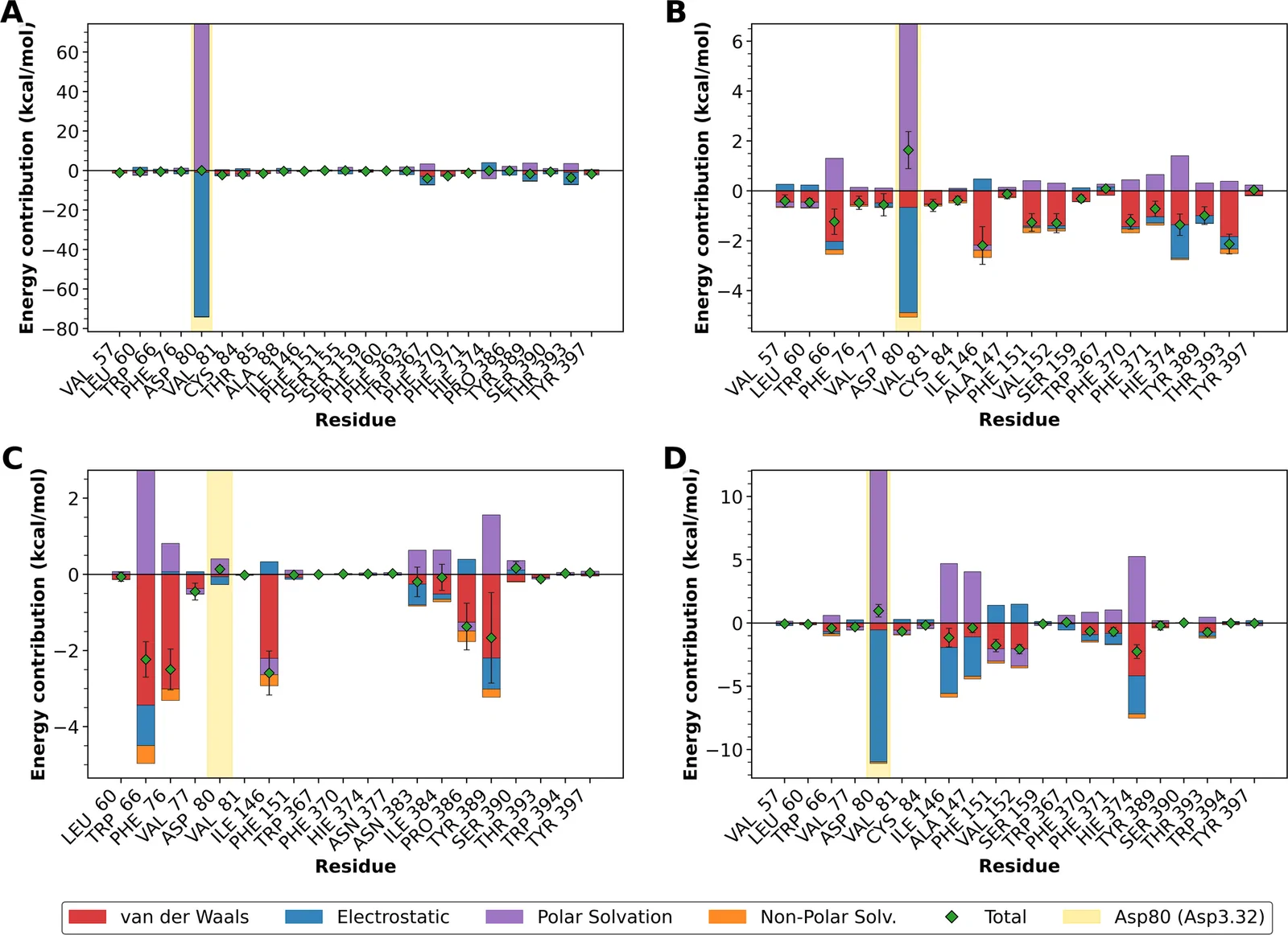
Per-residue MM-GBSA energy decomposition for the four simulated complexes computed over the final 50 ns of the 200 ns MD trajectory (1,000 snapshots). **(A)** Reference ligand 8NU. **(B)** DB08010. **(C)** DB12426. **(D)** DB18088

Approximate relative binding energetics were estimated using MM-GBSA within the same computational framework for all four systems (Table 7; Fig. 3). The reference ligand 8NU yielded the most favourable overall binding free energy (ΔG_bind_ = − 64.40 ± 5.00 kcal/mol), driven primarily by van der Waals contributions (ΔE_vdW_ = − 56.88 ± 3.22 kcal/mol).

DB08010 exhibited the second-most favourable binding free energy (ΔG_bind_ = − 46.78 ± 3.68 kcal/mol), with the strongest van der Waals contribution of all compounds (ΔE_vdW_ = − 58.39 ± 3.10 kcal/mol). DB18088 (ΔG_bind_ = − 35.15 ± 3.72 kcal/mol) and DB12426 (ΔG_bind_ = − 34.50 ± 4.05 kcal/mol) showed comparable overall binding free energies. DB18088 exhibited the most favourable electrostatic contribution (ΔE_ele_ = − 29.86 ± 7.37 kcal/mol) but also the highest polar solvation penalty (ΔG_pol_ = + 52.05 ± 7.60 kcal/mol).

Per-residue energy decomposition identified Asp80 as the primary electrostatic interaction site across all systems. Although Asp80 forms the conserved ionic interaction characteristic of aminergic GPCR ligands, its net MM-GBSA per-residue contribution remained slightly positive (8NU: +0.06 kcal/mol; DB08010: +1.63 kcal/mol; DB18088: +0.97 kcal/mol; DB12426: +0.14 kcal/mol). This reflects the balance between favourable local electrostatic interactions and the associated polar desolvation penalty included in MM-GBSA decomposition, resulting in a slightly unfavourable net energetic contribution despite its critical role in ligand recognition. Secondary contact residues included Trp367 (8NU: −3.96 kcal/mol), Thr393 (8NU: −3.64 kcal/mol; DB08010: −2.13 kcal/mol), Tyr389 (8NU: −1.66 kcal/mol; DB12426: −1.67 kcal/mol), Ile146 (DB08010: −2.19 kcal/mol; DB12426: −2.59 kcal/mol; DB18088: −1.16 kcal/mol), His374 (DB08010: −1.35 kcal/mol; DB18088: −2.26 kcal/mol), and Trp66 (DB08010: −1.24 kcal/mol; DB12426: −2.23 kcal/mol). Accordingly, MM-GBSA was used to provide approximate relative energetic estimates for comparing ligands within the same computational framework.

**Table 7:** MM-GBSA binding free energy components (mean ± SD, kcal/mol) computed over 1,000 snapshots sampled from the final 50 ns of the 200 ns MD trajectory

Component	8NU	DB08010	DB12426	DB18088
ΔG_bind_ (kcal/mol)	−64.40 ± 5.00	−46.78 ± 3.68	−34.50 ± 4.05	−35.15 ± 3.72
ΔE_vdW_ (kcal/mol)	−56.88 ± 3.22	−58.39 ± 3.10	−52.03 ± 4.46	−51.41 ± 4.03
ΔE_ele_ (kcal/mol)	−11.45 ± 6.96	−15.66 ± 3.65	−13.34 ± 5.17	−29.86 ± 7.37
ΔG_pol_ (kcal/mol)	+ 11.12 ± 4.39	+ 34.75 ± 2.79	+ 37.32 ± 5.35	+ 52.05 ± 7.60
ΔG_nonpol_ (kcal/mol)	−7.19 ± 0.31	−7.49 ± 0.28	−6.44 ± 0.43	−5.93 ± 0.37

ΔG_bind_ = total binding free energy; ΔE_vdW_ = van der Waals energy; ΔE_ele_ = electrostatic energy; ΔG_pol_ = polar solvation (GB) energy; ΔG_nonpol_ = non-polar solvation energy. 8NU is included as the co-crystallised reference ligand

### Model validation and robustness assessment

Three complementary analyses confirmed that the model captured genuine structure–activity signal rather than statistical artefact, and quantified the boundary of its reliable applicability. To exclude chance correlations, classifiers retrained on randomly permuted labels achieved a mean AUC of 0.461 ± 0.039 (indistinguishable from random), whereas the original XGBoost classifier achieved AUC = 0.881 under matched 5-fold stratified cross-validation, exceeding the permuted distribution by a z-score of 10.64 (*p* < 0.001; Figure S7).

The chemical-space gap between training and screening libraries was substantial. The curated ChEMBL training set contained 623 unique Bemis–Murcko scaffolds against 5,094 in DrugBank, with only 73 frameworks shared between the two (1.4% overlap). t-SNE embedding initialised from PCA components explaining 38.0% of descriptor variance reflected this scaffold divergence, with limited spatial overlap between the training and screening compounds (Figure S8A–B). Despite this divergence, applicability-domain analysis indicated that 293 of the 308 scaffold-split test compounds (95.1%) fell within the model’s reliable prediction range (threshold δ = 68.15); the 15 compounds outside this boundary represented structurally isolated scaffolds at the periphery of the training distribution (Figure S9).

## Discussion

### Chemical novelty and extrapolation in virtual screening

This study’s primary contribution is methodological. Specifically, the novelty lies in an interpretable, consensus-based prioritisation strategy that combines Bemis–Murcko scaffold-split-validated ensemble learning, SHAP interpretation, DrugBank virtual screening, GNINA docking, molecular dynamics simulation, and MM-GBSA analysis as complementary evidence streams, rather than in the development of any individual algorithm. The discovery of novel GPCR ligands remains constrained by the limited chemical space represented in historical bioactivity datasets used for model development. Models trained on such data struggle when applied to libraries containing structurally unfamiliar compounds. This study addresses the challenge of maintaining accuracy when most of the scaffolds in a screening library are absent from the training set. The integrated multi-stage pipeline mitigates this risk by combining ligand-based and structure-based evidence rather than relying on a single approach.

Chemical space analysis quantified the extent of this extrapolation. Among 5,094 unique Bemis–Murcko scaffolds identified in DrugBank, 98.6% were absent from the curated ChEMBL dataset used for model development. Consequently, most predictions were generated in genuine extrapolation space. Standard cross-validation metrics cannot fully capture uncertainty at this distance from the training distribution, necessitating the integration of partly complementary evidence streams to ensure prospective reliability. Notably, two of the three final candidates, Roginolisib (DB18088) and DB08010 (experimental), fall outside the model applicability domain (kNN distances: 70.09 and 97.00 respectively), while Bitopertin (DB12426) falls within it (kNN distance: 62.93; AD threshold δ = 68.15). For the two extrapolative candidates in particular, the multi-stage consensus pipeline rather than the ligand-based model alone carries the primary evidential weight for these predictions.

### Model validity and mechanistic interpretation

Ligand-based machine learning and structure-based docking capture distinct physicochemical determinants. Machine learning predicts IC₅₀-derived bioactivity from statistical relationships learned across experimentally characterised compounds, whereas docking evaluates the geometric and energetic compatibility of a ligand pose with a receptor structure using an empirical scoring function. The near-zero Pearson correlation (*r* = 0.090, *p* = 0.37, *n* = 100) between ML-predicted potencies and docking scores suggests that the two approaches capture complementary aspects of ligand recognition.

The low correlation may reflect genuine complementarity, but it may also arise from differences in scoring functions, the use of a single receptor conformation during docking, uncertainties in ligand protonation and binding pose prediction, and the limited correspondence between ligand-based bioactivity predictions and structure-based affinity estimates. Consequently, agreement between the two methods was interpreted as convergent computational evidence rather than definitive proof of binding, providing a more conservative basis for consensus filtering prior to molecular dynamics simulation. Beyond this complementarity, the ML component itself shows internal validity: Y-randomisation (z-score = 10.64, *p* < 0.001) and applicability domain analysis (95.1% of scaffold-split test compounds within the AD threshold) suggest the underlying models encode genuine structure–activity relationships.

Scaffold-split validation provided a stringent test of model generalisation. The Random Forest classifier achieved an AUC of 0.906 ± 0.016 (95% CI: 0.873–0.936), with the soft-voting ensemble attaining an AUC of 0.901 ± 0.017 and *R*² = 0.606 ± 0.043 for pIC₅₀ regression. The residual RMSE of 0.749 log units reflects, in part, the inter-assay heterogeneity inherent to aggregated ChEMBL data: a known noise floor for public-domain GPCR datasets that single-platform screening campaigns could in principle reduce, were such data available for DRD2. Performance decreased modestly relative to random-split cross-validation, consistent with the reduced structural overlap between training and test sets.

Generalisation was further supported by an independent external validation set constructed from PubChem BioAssay, comprising 247 compounds confirmed absent from the ChEMBL training set by canonical SMILES matching; the model achieved a AUC of 0.954 and an accuracy of 0.899 on this external set, comparable to and numerically higher than internal scaffold-split performance. Although substantial overlap between public bioactivity resources limited the size of the genuinely independent external subset, these results argue against simple memorisation of ChEMBL training compounds. Algorithm performance was compared using bootstrapped confidence intervals rather than a formal paired statistical test; the substantial overlap in these intervals across Random Forest, XGBoost, and LightGBM for both classification AUC and regression R² suggests comparable performance, though a paired significance test across repeated cross-validation folds would provide more rigorous confirmation.

SHAP analysis provided a chemically interpretable view of the structural motifs driving model predictions. The five top-ranked Morgan fingerprint bits decoded to nitrogen-containing substructural motifs: aromatic amines (Morgan_1295, 4.16×; Morgan_1647, 4.11×), a tertiary amine in an aromatic context (Morgan_231, 2.88×), a diamine linker (Morgan_1508, 1.69×), and a simple tertiary amine (Morgan_407, 1.66×). These chemically plausible DRD2-associated substructures are consistent with the conserved ionic interaction between protonated amines and Asp3.32 that underpins recognition of many DRD2 ligands. Because Morgan fingerprint bits encode local two-dimensional substructural environments rather than complete pharmacophore models, the SHAP analysis identifies chemically relevant substructures rather than reconstructing canonical DRD2 pharmacophores. Furthermore, SHAP values represent model-attributed feature importance rather than direct mechanistic evidence of receptor binding. Accordingly, these findings provide a biologically plausible explanation for the model’s predictions rather than causal validation of the underlying binding mechanism.

Physicochemical descriptors contributed independently to model performance. BCUT2D_LOGPHI, an eigenvalue-weighted descriptor reflecting the distribution of atomic lipophilicity, emerged as the most influential variable. SHAP dependence analysis identified threshold-like behaviour at approximately 2.28 units: below this value, molecules were consistently disfavoured; above it, predicted activity increased. NumAromaticRings modulated this relationship, indicating the joint role of ring count and lipophilicity in satisfying the hydrophobic demands of the orthosteric cavity.

While Morgan fingerprints alone achieved marginally higher scaffold-split classification accuracy than the full feature set (AUC = 0.910 vs. 0.901), regression performance required the combined descriptor set. Models using both fingerprints and physicochemical descriptors predicted continuous potency substantially better than fingerprints alone (*R*² = 0.606 vs. 0.578). Ranking compounds by predicted pIC₅₀ requires global information (lipophilicity, size, and electronic distribution) that fingerprints do not capture adequately across diverse scaffolds.

### Binding modes of prioritised candidates

Three DrugBank candidates were prioritised for molecular dynamics simulation: Bitopertin (DB12426), DB08010, and Roginolisib (DB18088). None of these compounds has reported experimental DRD2 activity; Bitopertin is an investigational GlyT1 inhibitor studied in schizophrenia and obsessive-compulsive disorder trials, DB08010 is an experimental stilbene-class oxindole derivative evaluated in virtual screening studies against MAPK10 (JNK3), and Roginolisib is an investigational PI3Kδ inhibitor.

All structural analyses were anchored to a single inactive-state receptor conformation (PDB: 6CM4); given that GPCRs adopt multiple functionally distinct states, the simulated geometries sample only one slice of the receptor’s accessible conformational landscape. Furthermore, a single 200 ns molecular dynamics trajectory was performed for each receptor–ligand complex without independent replicate simulations. Consequently, the reported conformational dynamics represent observations from individual exploratory trajectories rather than statistically reproducible estimates of ligand behaviour. The binding behaviours reported in this subsection therefore represent in silico predictions of potential DRD2 engagement that warrant experimental validation. In addition, all molecular dynamics simulations were performed in an explicit TIP3P water box without an embedded lipid bilayer. For a transmembrane GPCR such as DRD2, the absence of a membrane environment may influence transmembrane helix packing, orthosteric pocket geometry, solvent exposure, receptor dynamics, and ligand stability relative to the native physiological environment. Consequently, the RMSD, SASA, hydrogen-bond metrics, PCA conformational sampling, and MM-GBSA binding free energies reported below should be interpreted as comparative computational estimates within the same simulation framework rather than as quantitative measures of physiological binding affinity.

DB12426 adopted a binding geometry consistent with classical orthosteric engagement, with a nitrogen-containing moiety positioned near Asp3.32. However, molecular dynamics did not support stable pocket burial. The ligand exhibited the highest active-site RMSD of the three candidates (2.19 ± 0.45 Å) and a mean solvent-accessible surface area 75% greater than the reference ligand 8NU (798 vs. 455 Å²). Polar contact with Asp3.32 appeared insufficient to anchor the ligand deeply within the cavity without complementary hydrophobic packing.

The simulated DB08010 complex exhibited distinct thermodynamics. The ligand delivered the strongest van der Waals interaction of any simulated compound (Δ*E*_vdW_ = − 58.39 kcal/mol) and maintained stable pocket occupancy throughout the trajectory (RMSD = 1.81 ± 0.27 Å). A substantial polar solvation penalty (+ 34.75 kcal/mol) reflected the desolvation cost of burying polar groups, yet the geometry remained stable. These energetic terms, and those reported for the remaining candidates, derive from implicit-solvent simulations conducted in the absence of an explicit lipid bilayer; because membrane interactions are known to modulate orthosteric-pocket dynamics in GPCRs, the values are best interpreted as relative rankings within the simulated series rather than physiological affinities. As a compound currently characterised as a kinase inhibitor, DB08010 warrants experimental follow-up to determine whether this geometric complementarity extends to measurable DRD2 engagement.

DB18088 occupied an intermediate thermodynamic position. It showed the tightest predicted binding-site confinement among the candidates, with an active-site RMSD of 1.90 ± 0.27 Å and a PCA hull area of 433 Å² (− 48.8% relative to 8NU), suggesting that the ligand stabilises a narrower range of receptor conformations, potentially reflecting deeper burial within the orthosteric cavity or greater molecular rigidity relative to the reference ligand. DB18088 also exhibited the largest electrostatic contribution among the candidates (Δ*E*_ele_ = − 29.86 kcal/mol), but also the highest polar solvation penalty (+ 52.05 kcal/mol). Consistent with the per-residue MM-GBSA decomposition, the conserved Asp80 interaction contributes to ligand recognition through favourable local electrostatic interactions; however, its net residue contribution remains slightly positive because these interactions are offset by the associated polar desolvation penalty. These opposing forces yield a net computed binding free energy of − 35.15 kcal/mol. The high desolvation cost is consistent with DB18088’s large polar surface area, suggesting that several polar groups are not optimally accommodated within the receptor environment. Taken together, these computed thermodynamics distinguish three distinct binding profiles. MM-GBSA remains an approximation that incompletely captures entropic contributions; the final arbiter of DRD2 engagement will be the radioligand binding assay.

### Pharmacological context of final candidates

Beyond their predicted binding geometries, the translational relevance of the prioritised candidates depends on pharmacological properties beyond docking and molecular dynamics compatibility. Roginolisib (DB18088) is an investigational PI3Kδ inhibitor whose physicochemical properties suggest limited CNS penetration, potentially restricting its suitability for a CNS-targeted DRD2 indication. Its location outside the model applicability domain further increases uncertainty in the machine-learning prediction despite favourable structure-based results. DB08010 likewise lies outside the applicability domain and contains structural alerts identified by PAINS/Brenk filters, indicating that its predicted activity should be interpreted cautiously pending experimental validation. In contrast, Bitopertin (DB12426), a GlyT1 inhibitor previously investigated clinically for schizophrenia and obsessive-compulsive disorder, lies within the applicability domain, indicating greater structural similarity to known DRD2 ligands and correspondingly lower prediction uncertainty. Given the established role of DRD2 signalling in schizophrenia, its predicted DRD2 engagement is of particular translational interest. Nevertheless, for all three compounds, computational evidence of geometric and energetic compatibility does not establish clinically relevant receptor engagement. Whether DRD2 binding occurs at therapeutically attainable free plasma or brain concentrations, and whether adequate CNS exposure, target selectivity, and acceptable safety profiles (including potential hERG/QT liability) can be achieved, remain questions requiring experimental pharmacokinetic, receptor-binding, and functional studies.

### Implications for GPCR virtual screening

This work highlights three methodological considerations likely to generalise beyond DRD2. Our results support scaffold-split validation as a more rigorous standard for prospective screening: the performance gap observed between cross-validation (AUC = 0.952) and scaffold-split evaluation (AUC = 0.906) illustrates how random-split validation can inflate performance estimates through structural leakage between training and test sets. Second, combining partly complementary methods reduces reliance on any single computational approach. The low correlation between ML and docking scores indicates that these methods capture different aspects of ligand recognition, and consensus filtering integrates these complementary lines of evidence during candidate prioritisation. Third, interpretability analysis verifies that models have learned chemically meaningful relationships. Recovering the Asp3.32 pharmacophore from bioactivity data without structural input supports the conclusion that prospective predictions are grounded in chemistry rather than statistical artefacts.

Molecular dynamics simulation adds a temporal dimension that docking alone cannot provide. Static poses do not indicate whether a compound remains stable, how much it moves, or how much surface area remains exposed to solvent. For structurally novel scaffolds divergent from the training distribution, this dynamic picture is particularly valuable. Together, machine learning, docking, molecular dynamics, and interpretability analysis form a framework that should generalise to other data-sparse GPCR targets, particularly where screening libraries extend beyond previously characterised chemical space.

## Conclusions

Combining ligand-based machine learning, structure-based docking, and molecular dynamics simulation provides a reproducible framework for prioritising chemically novel GPCR ligands. Interpretability analysis indicated that the Random Forest model recapitulated established DRD2 pharmacophore elements directly from heterogeneous bioactivity data, specifically nitrogen-containing motifs consistent with the canonical Asp3.32 salt-bridge interaction. The integration of physicochemical descriptors with structural fingerprints was necessary for accurate potency prediction, highlighting the importance of global molecular properties in regression tasks.

The low correlation between ML predictions and docking scores indicates that the two approaches capture partly complementary information, supporting their integration within a consensus screening strategy. Application of this pipeline to DrugBank computationally prioritised three candidate compounds; Roginolisib (DB18088), DB08010, and Bitopertin (DB12426) which exhibited distinct binding geometries and dynamic behaviour during exploratory molecular dynamics simulations. While none of the candidates has reported experimental DRD2 activity, they represent computationally prioritised hypotheses that warrant experimental validation through radioligand binding and functional assays to determine whether the predicted receptor engagement translates to measurable biological activity.

Scaffold-split validation, independent PubChem BioAssay external validation, Y-randomisation (z = 10.64, *p* < 0.001), and applicability-domain analysis support the robustness of the machine-learning component within its defined domain of applicability. On the non-overlapping PubChem external set, the ensemble retained strong classification performance (AUC = 0.954; accuracy = 0.899), although regression performance was more moderate (R² = 0.515; MAE = 0.597 pIC_50_), consistent with the expected quantitative noise in public bioactivity data. The recovery of nine known DRD2-active drugs among the top 100 docked DrugBank candidates further demonstrates enrichment of established DRD2 pharmacophores. Nevertheless, the molecular dynamics simulations were performed as single exploratory aqueous-phase trajectories using a single receptor conformation and should therefore be interpreted comparatively rather than as definitive evidence of physiological binding behaviour.

## Supplementary Information

Below is the link to the electronic supplementary material.

Supplementary Material 1

## Data Availability

All source code, trained machine-learning models, analysis notebooks, and the curated ChEMBL datasets used for model development and screening results are available at: https://github.com/sphamtambo/drd2-qsar-repurposing. The repository also includes the scripts required to reproduce the ChEMBL data retrieval and curation pipeline. The DrugBank screening library is not redistributed because it is subject to the DrugBank non-commercial licence. Users wishing to reproduce the virtual screening should independently obtain the DrugBank compound library from the official DrugBank website and place it in the directory specified in the repository documentation before executing the screening workflow.
