# Supplementary material for: An interpretable consensus pipeline for candidate dopamine D2 receptor ligands
Source: In Silico Pharmacol. 2026 Sep 24;14(3):251. doi: 10.1007/s40203-026-00749-7 (PMC13612801; doi:10.1007/s40203-026-00749-7)
Supplement: Supplementary file 1 [file 40203_2026_749_MOESM1_ESM.docx]

**Supplementary Material**

**An Interpretable Consensus Pipeline for Candidate Dopamine D2 Receptor Ligands**

**Sphamandla Mtambo¹, Hezekiel M. Kumalo^2^, Cornelius Cano Ssemakalu¹,**

¹Cell Biology and Drug Discovery Research Group, Department of Natural Sciences, Faculty of Applied and Computer Sciences, Vaal University of Technology, Vanderbijlpark, South Africa

^2^Drug Research and Innovation Unit, Discipline of Medical Biochemistry, School of Laboratory Medicine and Medical Science, University of KwaZulu-Natal, Durban, South Africa

**Author Emails:** Sphamandla Mtambo: sphamandlam@vut.ac.za, Hezekiel H. Kumalo; kumaloh@ukzn.ac.za

***Correspondence:** Cornelius Cano Ssemakalu: corneliuss@vut.ac.za, Sphamandla Mtambo: sphamandlam@vut.ac.za,

**Sphamandla Mtambo^1^** - ORCID ID: 0000-0003-3444-7073

**Hezekiel Kumalo^2^** - ORCID ID: 0000-0002-4037-1549

**Cornelius Cano Ssemakalu^1^** - ORCID ID: [0000-0003-3895-2018](https://orcid.org/0000-0003-3895-2018)

**Table S1.** Final hyperparameters selected by Optuna Bayesian optimisation (500 trials, TPE sampler, MedianPruner with 5 warm-up steps) for all six model-task combinations. Classification models were optimised to maximise AUC-ROC; regression models were optimised to maximise R², both evaluated using 20-fold scaffold GroupKFold cross-validation within the training set. Parameter ranges: n_estimators (100–1,000), max_depth (5–20), learning_rate (0.01–0.15, log-uniform), subsample (0.6–1.0), colsample_bytree (0.15–0.5), colsample_bylevel (0.3–1.0), min_child_weight (1–10), gamma (0–0.5), reg_alpha (0–2.0), reg_lambda (0–2.0). RF-specific: min_samples_split (2–20), min_samples_leaf (1–10), max_features (sqrt, log2, or float 0.1–0.5), bootstrap (True/False). LightGBM-specific: num_leaves (20–200), min_child_samples (5–50). — = parameter not applicable to that algorithm.

| **Hyperparameter** | **RF** | **XGBoost** | **LightGBM** | **RF** | **XGBoost** | **LightGBM** |
| --- | --- | --- | --- | --- | --- | --- |
|  | **Classification** | | | **Regression** | | |
| **n_estimators** | 461 | 872 | 493 | 331 | 399 | 232 |
| **max_depth** | 27 | 10 | 23 | 30 | 19 | 14 |
| **num_leaves** | — | — | 32 | — | — | 141 |
| **learning_rate** | — | 0.012 | 0.016 | — | 0.066 | 0.076 |
| **min_samples_split** | 11 | — | — | 7 | — | — |
| **min_samples_leaf** | 3 | — | — | 1 | — | — |
| **min_child_weight** | — | 1 | — | — | 2 | — |
| **min_child_samples** | — | — | 8 | — | — | 18 |
| **max_features** | sqrt | — | — | 0.30 | — | — |
| **subsample** | — | 0.820 | 0.911 | — | 0.745 | 0.820 |
| **colsample_bytree** | — | 0.183 | 0.266 | — | 0.346 | 0.292 |
| **colsample_bylevel** | — | 0.702 | — | — | 0.327 | — |
| **gamma** | — | 0.058 | — | — | 0.033 | — |
| **reg_alpha** | — | 0.545 | 0.082 | — | 1.990 | 0.005 |
| **reg_lambda** | — | 1.051 | 0.063 | — | 0.138 | 0.243 |
| **bootstrap** | True | — | — | — | — | — |
| **class_weight** | bal_sub | — | — | — | — | — |

**Figure S1A.** ROC curves for RF, XGBoost, LightGBM, and soft-voting ensemble on scaffold-stratified held-out test set (n = 308). AUC values in legend are single-split point estimates; bootstrapped mean ± SD reported in Table 1 (main text). Dashed diagonal = random baseline (AUC = 0.5).

**Figure S1B**. Precision-recall curves for RF, XGBoost, LightGBM, and soft-voting ensemble on scaffold-stratified held-out test set (n = 308). Average precision (AP): RF 0.877 ± 0.025, XGBoost 0.871 ± 0.024, LightGBM 0.876 ± 0.023, Ensemble 0.872 ± 0.025. Dashed horizontal line = baseline AP at active class prevalence in test set.

**Figure S1C.** Probability calibration curves for RF, XGBoost, LightGBM, and soft-voting ensemble on scaffold-stratified held-out test set (n = 308). Predicted probabilities binned into ten equal-width intervals; observed positive class frequency plotted against mean predicted probability. Dashed diagonal = perfect calibration. Brier scores: RF 0.131 ± 0.009, XGBoost 0.136 ± 0.013, LightGBM 0.137 ± 0.015, Ensemble 0.128 ± 0.012.

**Figure S1D.** Confusion matrix for Random Forest classifier on scaffold-stratified held-out test set (n = 308). Accuracy = 0.811 ± 0.023 (95% CI: 0.766–0.857). Active-class precision = 0.832 ± 0.037; recall = 0.694 ± 0.041; F1 = 0.756 ± 0.032.

**Figure S2A**. Predicted versus actual pIC₅₀ scatter plot for RF, XGBoost, LightGBM, and ensemble regression models on scaffold-stratified held-out test set (n = 308). Dashed red line = perfect prediction. Ensemble R² = 0.606 (RF 0.603, XGBoost 0.604, LightGBM 0.568).

**Figure S2B.** Residual plot for ensemble regression model on scaffold-stratified held-out test set (n = 308). Residuals (actual minus predicted pIC₅₀) plotted against predicted pIC₅₀. Dashed red horizontal line = zero residual. Ensemble RMSE = 0.749 pIC₅₀ units.

**Table S2.** Top five Morgan fingerprint bits by mean |SHAP| from the Random Forest classifier (scaffold-split AUC = 0.906; TreeSHAP on scaffold-stratified test set, n = 308). SMARTS decoded via RDKit bitInfo at radius 2. Enrichment = (% actives with bit) / (% inactives with bit).

| **Bit ID** | **Mean \|SHAP\|** | **SMARTS Pattern** | **% Actives** | **% Inactives** | **Enrichment** |
| --- | --- | --- | --- | --- | --- |
| Morgan_1295 | 0.0183 | [#7]-[#6](:[#6]):[#6] | 46.1% | 11.1% | 4.16× |
| Morgan_231 | 0.0126 | [#6]-[#6]-[#7](-[#6](:[#6]):[#6])-[#6]-[#6] | 50.0% | 17.4% | 2.88× |
| Morgan_1508 | 0.0116 | [#7]-[#6]-[#6]-[#7](-[#6])-[#6] | 54.5% | 32.2% | 1.69× |
| Morgan_1647 | 0.0110 | [#7]-[#6](:[#6]:[#6]:[#6]):[#6] | 42.0% | 10.2% | 4.11× |
| Morgan_407 | 0.0110 | [#6]-[#7](-[#6])-[#6] | 55.0% | 33.0% | 1.66× |

**Figure S3A.** Mean |SHAP value| for top 20 features of Random Forest classifier (Full feature set; scaffold-stratified test set, n = 308). Features ranked in descending order by mean absolute SHAP value. BCUT2D_LOGPHI = 0.0261; Morgan_1295 = 0.0183; Morgan_231 = 0.0126; Morgan_1508 = 0.0116; Morgan_1647 = 0.0110; Morgan_407 = 0.0110.

**Figure S3B.** Random Forest built-in mean decrease in impurity (MDI) importance for top 15 features (Full feature set; scaffold-stratified test set, n = 308). Features ranked in descending order by MDI score. BCUT2D_LOGPHI ranks first by both MDI and mean |SHAP|.

**Figure S3C.** SHAP beeswarm summary plot for Random Forest classifier (Full feature set; scaffold-stratified test set, n = 308). Each dot = one compound. Horizontal position = SHAP value (impact on probability of active prediction). Colour = relative feature value (red = high, blue = low). Features ranked by mean |SHAP| in descending order.

**Figure S3D.** SHAP dependence plot for BCUT2D_LOGPHI (mean |SHAP| = 0.0261; top-ranked feature). Colour = HeavyAtomCount (SHAP interaction variable). X-axis = BCUT2D_LOGPHI feature value. Y-axis = SHAP value.

**Figure S3E.** SHAP dependence plot for Morgan_1295 (mean |SHAP| = 0.0183; SMARTS:[#7]-[#6](:[#6]):[#6]; aromatic amine). Colour = BertzCT (SHAP interaction variable). X-axis = bit presence (0 = absent, 1 = present). Y-axis = SHAP value. Enrichment = 4.16× (46.1% actives vs 11.1% inactives).

**Figure S3F.** SHAP dependence plot for Morgan_231 (mean |SHAP| = 0.0126; SMARTS: [#6]-[#6]-[#7](-[#6](:[#6]):[#6])-[#6]-[#6]; tertiary amine in aromatic context). Colour = BertzCT (SHAP interaction variable). X-axis = bit presence (0 = absent, 1 = present). Y-axis = SHAP value. Enrichment = 2.88× (50.0% actives vs 17.4% inactives).

**Figure S3G.** SHAP dependence plot for Morgan_1508 (mean |SHAP| = 0.0116; SMARTS: [#7]-[#6]-[#6]-[#7](-[#6])-[#6]; diamine linker). Colour = RingCount (SHAP interaction variable). X-axis = bit presence (0 = absent, 1 = present). Y-axis = SHAP value. Enrichment = 1.69× (54.5% actives vs 32.2% inactives).

**Figure S3H.** SHAP dependence plot for Morgan_1647 (mean |SHAP| = 0.0110; SMARTS: [#7]-[#6](:[#6]:[#6]:[#6]):[#6]; extended aromatic amine). Colour = RingCount (SHAP interaction variable). X-axis = bit presence (0 = absent, 1 = present). Y-axis = SHAP value. Enrichment = 4.11× (42.0% actives vs 10.2% inactives).

**Figure S4.** Spearman rank correlation heatmap for the top 50 most important features (ranked by mean |SHAP|). Red = positive correlation; blue = negative; white = near-zero. Features include BCUT2D descriptors, Morgan fingerprint bits, and physicochemical descriptors.

**Figure S5.** Distribution of GNINA CNN pose quality scores across 100 compounds advanced to structure-based docking from DrugBank virtual screening. Red dashed line = median CNN score (0.58). X-axis = CNN score (0–1); Y-axis = frequency.

**Table S3.** Physicochemical properties and applicability domain status of the three final MD simulation candidates.

| **Property** | **Roginolisib (DB18088)** | **DB08010** | **Bitopertin (DB12426)** |
| --- | --- | --- | --- |
| **DrugBank ID** | DB18088 | DB08010 | DB12426 |
| **Generic name** | Roginolisib | Experimental compound | Bitopertin |
| **DrugBank status** | Investigational | Experimental | Investigational |
| **Primary target** | PI3Kδ | MAPK10 (JNK3) | GlyT1 |
| **Therapeutic indication** | Oncology | Not established | Schizophrenia and OCD |
| **SMILES** | O=C(c1nn(-c2ccc(CN3CCOCC3)cc2)c2c1CS(=O)(=O)c1c(F)cccc1-2)N1CCOCC1 | O=C1/C(=N\O)c2c(/C=C/c3ccccc3)cccc2N1Cc1cc(F)cc2c1OCOC2 | C[C@H](Oc1ccc(S(C)(=O)=O)cc1C(=O)N1CCN(c2ncc(C(F)(F)F)cc2F)CC1)C(F)(F)F |
| **MW (g/mol)** | 526.59 | 430.44 | 543.46 |
| **LogP** | 2.27 | 4.59 | 3.94 |
| **TPSA (Å²)** | 93.97 | 71.36 | 79.81 |
| **HBD** | 0 | 1 | 0 |
| **HBA** | 7 | 5 | 6 |
| **Rotatable bonds** | 4 | 4 | 5 |
| **Charge state** | Neutral | Neutral | Neutral |
| **PAINS/Brenk flag** | None | Imine, oxime, O–N single bond, stilbene | None |
| **CNS penetration (rule-of-thumb)** | Unfavourable (MW > 450) | Unfavourable (LogP > 4) | Unfavourable (MW > 450) |
| **AD distance** | 70.09 | 97.00 | 62.93 |
| **AD status** | Outside AD | Outside AD | Within AD |

*MW, molecular weight; LogP, octanol-water partition coefficient (RDKit); TPSA, topological polar surface area; HBD, hydrogen bond donors; HBA, hydrogen bond acceptors. All physicochemical properties computed using RDKit (v2023.09.1). Charge states reflect canonical SMILES as retrieved from DrugBank v5.1.13 without explicit pH reassignment. PAINS/Brenk flag, structural alerts identified using the combined RDKit PAINS and Brenk filter catalogues, consistent with the methodology applied during ChEMBL training-set curation. CNS penetration estimated using simplified rule-of-thumb criteria (MW < 450, TPSA < 90, LogP 1-4, HBD ≤ 2); this is a heuristic indicator, not a validated blood-brain-barrier permeability model. AD distance, mean kNN Euclidean distance in standardised feature space (k = 5; threshold δ = 68.15).*

**Table S4.** Established DRD2-active drugs recovered among 100 compounds selected for docking. All nine compounds assigned ensemble activity probabilities ≥ 0.976 prior to docking. ML rank = rank by ensemble probability among 100 docked compounds. Affinity = GNINA minimized binding affinity (kcal/mol); CNN Score = GNINA CNN pose quality score.

| **ID** | **Drug** | **Pred. pIC₅₀** | **Ens. Prob.** | **Affinity** | **CNN Score** | **Known DRD2 Mechanism** |
| --- | --- | --- | --- | --- | --- | --- |
| DB09226 | Brilaroxazine | 8.41 | 0.999 | −9.060 | 0.386 | Partial agonist (Investigational) |
| DB00623 | Fluphenazine | 8.56 | 0.999 | −10.000 | 0.578 | Antagonist (FDA-approved) |
| DB00831 | Trifluoperazine | 8.01 | 0.995 | −9.869 | 0.444 | Antagonist (FDA-approved) |
| DB00850 | Perphenazine | 9.00 | 0.993 | −9.311 | 0.615 | Antagonist (FDA-approved) |
| DB00933 | Mesoridazine | 7.62 | 0.991 | −9.367 | 0.740 | Antagonist (FDA-approved) |
| DB00477 | Chlorpromazine | 7.88 | 0.990 | −7.913 | 0.506 | Antagonist (FDA-approved) |
| DB00433 | Prochlorperazine | 7.99 | 0.986 | −9.121 | 0.630 | Antagonist (FDA-approved) |
| DB01063 | Acetophenazine | 8.53 | 0.977 | −9.009 | 0.637 | Antagonist (FDA-approved) |
| DB01621 | Pipotiazine | 8.63 | 0.976 | −9.376 | 0.443 | Antagonist (FDA-approved) |

**Figure S6.** Ensemble-predicted pIC₅₀ versus GNINA minimized binding affinity (kcal/mol) for top 100 compounds advanced to docking from DrugBank virtual screening. Each point = one compound. Pearson correlation coefficient r = 0.090. Reference ligand 8NU affinity = −10.80 kcal/mol.

**Table S5.** Ligand stability metrics during 200 ns MD simulations

| **Metric** | **8NU** | **DB18088** | **DB08010** | **DB12426** |
| --- | --- | --- | --- | --- |
| Ligand RMSD (Å), mean ± SD | 0.475 ± 0.150 | 1.149 ± 0.223 | 1.505 ± 0.550 | 2.174 ± 0.370 |
| Ligand RMSD final frame (Å) | 0.640 | 1.329 | 0.972 | 2.804 |
| Hydrogen-bond occupancy (%) | 100.0 | 98.7 | 92.1 | 98.9 |
| Mean hydrogen bonds | 7.326 | 3.110 | 2.370 | 3.442 |
| Ligand RMSF mean (Å) | 1.716 | 2.463 | 3.481 | 5.259 |
| Ligand RMSF maximum (Å) | 2.698 | 3.848 | 6.007 | 6.737 |

**Figure S7.** Y-randomization test for XGBoost classifier (Full feature set; identical hyperparameters; 5-fold CV; n = 100 permutation iterations). Orange histogram = AUC distribution from models trained on permuted activity labels (mean = 0.461). Blue dashed line = original model AUC (0.881). Red dotted line = chance level (0.500). Z-score = 10.64, p < 0.001.

**Figure S8A.** t-SNE visualization of training (ChEMBL, n = 1,247) and screening (DrugBank, n = 10,041) compounds. Initialised from PCA components explaining 38.0% of descriptor variance. Blue = ChEMBL training compounds; orange = DrugBank screening compounds.

**Figure S8B.** Bemis–Murcko scaffold Venn diagram. Training set (ChEMBL): 623 unique scaffolds from 1,247 curated compounds. DrugBank: 5,094 unique scaffolds from 10,041 compounds. Shared scaffolds: 73 (1.4% of DrugBank; 11.7% of training scaffolds). DrugBank-only scaffolds: 5,021 (98.6% of DrugBank).

**Figure S9.** Applicability domain (AD) analysis using kNN Euclidean distance in standardized feature space (k = 5; threshold = mean + 2σ of training kNN distances = 68.15). Each point = scaffold-stratified held-out test compound (n = 308). X-axis = mean kNN distance in standardized feature space. Y-axis = standardized residuals. Blue = within-AD (293/308; 95.1%); red = outside-AD (15/308; 4.9%). Red dashed vertical line = AD threshold (68.15). Dotted horizontal lines = ±2 SD residual bounds.

**Figure S10.** Comparison of crystallographic and docked binding poses for the reference ligand 8NU (Risperidone) at the DRD2 orthosteric site (PDB: 6CM4). (A) Crystallographic pose, extracted directly from the deposited 6CM4 coordinates prior to docking. (B) Docked pose, generated using the validated GNINA docking protocol (heavy-atom RMSD relative to the crystallographic pose = 1.428 Å). The redocked pose retained the key Asp80 anchoring interaction and major hydrophobic contacts within the binding pocket.
